# 1,7,8,9,10,10-Hexachloro-4-(thio­phen-2-ylmeth­yl)-4-aza­tricyclo­[5.2.1.0^2,6^]dec-8-ene-3,5-dione

**DOI:** 10.1107/S1600536811032788

**Published:** 2011-08-27

**Authors:** R. Manohar, M. Harikrishna, C. R. Ramanathan, M. SureshKumar, K. Gunasekaran

**Affiliations:** a CAS in Crystallography and Biophysics, University of Madras, Guindy Campus, Chennai 600025, India; b Department of Chemistry, Pondicherry University, Pondicherry 605014, India; c Centre for Bioinformatics, Pondicherry University, Pondicherry 605014, India

## Abstract

In the title compound, C_14_H_7_Cl_6_NO_2_S, the six-membered ring of the aza­tricyclo system has a boat conformation whereas the five-membered rings have an envelope conformation. The thio­phene ring and the ring of the succinimide moiety enclose a dihedral angle of 67.2 (1)°. The crystal packing is stabilized by weak inter­molecular C—H⋯O hydrogen bonds.

## Related literature

For the biological activity of cyclic imides, see: Duarte *et al.* (2006 ▶); Nakamura *et al.* (2006 ▶); Stefańska *et al.* (2010 ▶).
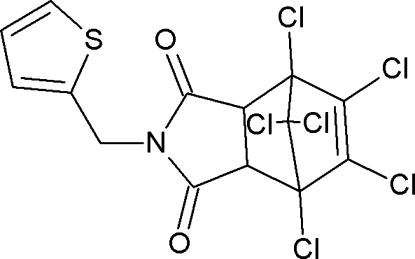


## Experimental

### 

#### Crystal data

C_14_H_7_Cl_6_NO_2_S
*M* *_r_* = 465.97
Tetragonal, 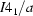

*a* = 23.8136 (10) Å
*c* = 12.6240 (9) Å
*V* = 7158.9 (7) Å^3^
*Z* = 16
Mo *K*α radiation
μ = 1.08 mm^−1^
*T* = 293 K
0.20 × 0.20 × 0.20 mm Is this OK?

#### Data collection

Xcalibur, Eos diffractometer
Absorption correction: multi-scan (*CrysAlis PRO*; Oxford Diffraction, 2010 ▶) *T*_min_ = 0.978, *T*_max_ = 0.984
8488 measured reflections
4156 independent reflections
2283 reflections with *I* > 2σ(*I*)
*R*_int_ = 0.032

#### Refinement

*R*[*F* ^2^ > 2σ(*F* ^2^)] = 0.037
*wR*(*F* ^2^) = 0.075
*S* = 0.82
4156 reflections
217 parameters
H-atom parameters constrained
Δρ_max_ = 0.31 e Å^−3^
Δρ_min_ = −0.31 e Å^−3^

### 

Data collection: *CrysAlis PRO* (Oxford Diffraction, 2010 ▶); cell refinement: *CrysAlis PRO*; data reduction: *CrysAlis PRO*; program(s) used to solve structure: *SHELXS97* (Sheldrick, 2008 ▶); program(s) used to refine structure: *SHELXL97* (Sheldrick, 2008 ▶); molecular graphics: *ORTEP-3* (Farrugia, 1997 ▶); software used to prepare material for publication: *SHELXL97* and *PLATON* (Spek, 2009 ▶).

## Supplementary Material

Crystal structure: contains datablock(s) I, global. DOI: 10.1107/S1600536811032788/bt5586sup1.cif

Structure factors: contains datablock(s) I. DOI: 10.1107/S1600536811032788/bt5586Isup2.hkl

Supplementary material file. DOI: 10.1107/S1600536811032788/bt5586Isup3.cml

Additional supplementary materials:  crystallographic information; 3D view; checkCIF report

## Figures and Tables

**Table 1:** Hydrogen-bond geometry (Å, °)

*D*—H⋯*A*	*D*—H	H⋯*A*	*D*⋯*A*	*D*—H⋯*A*
C2—H2⋯O2^i^	0.98	2.54	3.064 (3)	113
C6—H6⋯O2^i^	0.98	2.51	3.042 (3)	114

Symmetry code: (i) 
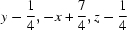
.

**Fig. 1.**
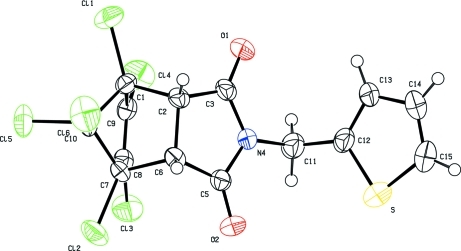
The ORTEP diagram of the compound with 30% probability displacement ellipsoids.

**Fig. 2.**
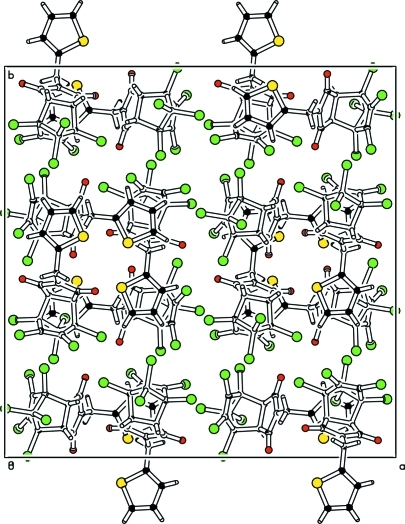
Packing diagram.

## supplementary crystallographic information

### Comment

Azatricyclo dec-8-ene 3,5 dione derivatives have anti bacterial and anti fungal activities with other important biological activities (Stefańska *et al.*, 2010).

In these structure, the six-membered ring of the norbornene moiety adopts a boat conformation whereas the two five-membered rings adopt envelope conformation.The fusion at atoms C6 and C2 is in *cis* conformation. The planarity around N4 and C3—N4 [1.38 (4) Å] and N4—C5 [1.38 (4) Å] reveals the partial double bond charater to facilitate the electron delocalization from one keto oxygen to other through N4. The crystal structure is stabilized by weak inter-molecular C-H···O interactions.

### Experimental

1-(thiophen-2-yl)methanamine (1 equiv) and 1,4,5,6,7,7-hexachloro-5-norbornene-2,3-dicarboxylic anhydride (1 equiv) were stirred at room temperature in dry ethyl acetate for 30 min. Ethyl acetate was removed under reduced pressure; the resulting residue was dissolved in toluene. To this reaction mixture was added acetyl chloride (5 equiv) and refluxed for 1 h. The reaction mixture was brought to room temperature and washed with aqueous Na2CO3 and dried over anhydrous Na2SO4. Filtered and concentrated under reduced pressure followed by silica gel column purification afforded the imide, 1,7,8,9,10,10-Hexachloro-4-(thiophen-2-yl-methyl)-4-azatricyclo[5.2.1.0^2,6^]dec-8-ene-3,5-dione, in 92% yield as colorless solid.

### Refinement

The hydrogen atoms   were positioned geometrically and refined using a riding model.

### Crystal data

**Table:**

C_14_H_7_Cl_6_NO_2_S	*F*(000) = 3712
*M**_r_* = 465.97	*D*_x_ = 1.729 Mg m^−^^3^
Tetragonal, *I*4_1_/*a*	Mo *K*α radiation, λ = 0.71073 Å
Hall symbol: -I 4ad	µ = 1.08 mm^−^^1^
*a* = 23.8136 (10) Å	*T* = 293 K
*c* = 12.6240 (9) Å	Tetragonal, colourless
*V* = 7158.9 (7) Å^3^	0.20 × 0.20 × 0.20 mm
*Z* = 16	

### Data collection

**Table:**

Oxford Diffraction Xcalibur Eos diffractometer	4156 independent reflections
Radiation source: fine-focus sealed tube	2283 reflections with *I* > 2σ(*I*)
graphite	*R*_int_ = 0.032
Detector resolution: 15.9821 pixels mm^-1^	θ_max_ = 29.2°, θ_min_ = 3.0°
ω scans	*h* = −29→16
Absorption correction: multi-scan (*CrysAlis PRO*; Oxford Diffraction, 2010)	*k* = −28→32
*T*_min_ = 0.978, *T*_max_ = 0.984	*l* = −15→17
8488 measured reflections	

### Refinement

**Table:**

Refinement on *F*^2^	Primary atom site location: structure-invariant direct methods
Least-squares matrix: full	Secondary atom site location: difference Fourier map
*R*[*F*^2^ > 2σ(*F*^2^)] = 0.037	Hydrogen site location: inferred from neighbouring sites
*wR*(*F*^2^) = 0.075	H-atom parameters constrained
*S* = 0.82	*w* = 1/[σ^2^(*F*_o_^2^) + (0.0329*P*)^2^] where *P* = (*F*_o_^2^ + 2*F*_c_^2^)/3
4156 reflections	(Δ/σ)_max_ = 0.001
217 parameters	Δρ_max_ = 0.31 e Å^−^^3^
0 restraints	Δρ_min_ = −0.31 e Å^−^^3^

### Special details

**Table:**

Geometry. All e.s.d.'s (except the e.s.d. in the dihedral angle between two l.s. planes) are estimated using the full covariance matrix. The cell e.s.d.'s are taken into account individually in the estimation of e.s.d.'s in distances, angles and torsion angles; correlations between e.s.d.'s in cell parameters are only used when they are defined by crystal symmetry. An approximate (isotropic) treatment of cell e.s.d.'s is used for estimating e.s.d.'s involving l.s. planes.
Refinement. Refinement of *F*^2^ against ALL reflections. The weighted *R*-factor *wR* and goodness of fit *S* are based on *F*^2^, conventional *R*-factors *R* are based on *F*, with *F* set to zero for negative *F*^2^. The threshold expression of *F*^2^ > σ(*F*^2^) is used only for calculating *R*-factors(gt) *etc*. and is not relevant to the choice of reflections for refinement. *R*-factors based on *F*^2^ are statistically about twice as large as those based on *F*, and *R*- factors based on ALL data will be even larger.

### Fractional atomic coordinates and isotropic or equivalent isotropic displacement parameters (Å^2^)

**Table:**

	*x*	*y*	*z*	*U*_iso_*/*U*_eq_	
C1	0.60327 (10)	1.16190 (10)	0.08398 (18)	0.0301 (6)	
C2	0.66296 (9)	1.13668 (9)	0.09872 (19)	0.0295 (6)	
H2	0.6864	1.1422	0.0358	0.035*	
C3	0.69158 (10)	1.15740 (10)	0.1978 (2)	0.0326 (6)	
C5	0.67499 (10)	1.06286 (10)	0.2282 (2)	0.0344 (6)	
C6	0.65133 (9)	1.07395 (9)	0.11968 (18)	0.0288 (6)	
H6	0.6690	1.0500	0.0660	0.035*	
C7	0.58607 (9)	1.07054 (9)	0.11446 (18)	0.0276 (5)	
C8	0.56310 (9)	1.10361 (10)	0.20743 (17)	0.0291 (6)	
C9	0.57323 (9)	1.15729 (10)	0.18980 (18)	0.0285 (6)	
C10	0.57402 (9)	1.11314 (11)	0.02405 (18)	0.0342 (6)	
C11	0.72133 (10)	1.11804 (11)	0.3724 (2)	0.0432 (7)	
H11A	0.7054	1.0897	0.4187	0.052*	
H11B	0.7116	1.1546	0.4009	0.052*	
C12	0.78385 (11)	1.11200 (10)	0.37121 (19)	0.0391 (7)	
C13	0.82275 (11)	1.15206 (11)	0.3454 (2)	0.0485 (8)	
H13	0.8140	1.1888	0.3269	0.058*	
C14	0.87777 (12)	1.13051 (13)	0.3504 (2)	0.0617 (9)	
H14	0.9094	1.1520	0.3355	0.074*	
C15	0.88049 (12)	1.07617 (13)	0.3788 (2)	0.0573 (8)	
H15	0.9137	1.0559	0.3857	0.069*	
N4	0.69675 (8)	1.11223 (8)	0.26664 (16)	0.0321 (5)	
O1	0.70632 (7)	1.20435 (7)	0.21805 (15)	0.0486 (5)	
O2	0.67518 (8)	1.01900 (7)	0.27599 (16)	0.0550 (5)	
S	0.81534 (3)	1.04915 (3)	0.40044 (6)	0.0562 (2)	
Cl1	0.60119 (3)	1.22738 (3)	0.02283 (6)	0.0557 (2)	
Cl2	0.55865 (3)	1.00296 (3)	0.09833 (6)	0.0508 (2)	
Cl3	0.53529 (3)	1.07338 (3)	0.31710 (6)	0.0528 (2)	
Cl4	0.56334 (3)	1.21203 (3)	0.27281 (6)	0.0553 (2)	
Cl5	0.50195 (3)	1.12482 (3)	0.00103 (6)	0.0524 (2)	
Cl6	0.60575 (3)	1.09588 (3)	−0.09771 (5)	0.0593 (2)	

### Atomic displacement parameters (Å^2^)

**Table:**

	*U*^11^	*U*^22^	*U*^33^	*U*^12^	*U*^13^	*U*^23^
C1	0.0295 (13)	0.0306 (13)	0.0303 (14)	0.0027 (11)	0.0006 (11)	0.0069 (11)
C2	0.0227 (12)	0.0304 (13)	0.0353 (14)	−0.0024 (11)	0.0064 (11)	0.0025 (11)
C3	0.0210 (12)	0.0312 (14)	0.0456 (17)	−0.0027 (12)	0.0021 (12)	−0.0004 (13)
C5	0.0246 (13)	0.0282 (14)	0.0502 (17)	0.0013 (11)	−0.0019 (12)	0.0016 (13)
C6	0.0233 (12)	0.0267 (12)	0.0363 (15)	0.0006 (11)	0.0046 (11)	−0.0074 (11)
C7	0.0259 (12)	0.0271 (13)	0.0300 (14)	−0.0056 (11)	−0.0008 (11)	−0.0045 (11)
C8	0.0221 (12)	0.0384 (14)	0.0269 (14)	−0.0004 (12)	0.0030 (10)	0.0015 (12)
C9	0.0239 (12)	0.0318 (14)	0.0298 (14)	0.0080 (12)	0.0016 (11)	−0.0028 (11)
C10	0.0250 (13)	0.0519 (16)	0.0258 (13)	0.0007 (13)	−0.0012 (11)	−0.0058 (12)
C11	0.0458 (16)	0.0434 (16)	0.0404 (17)	0.0009 (14)	−0.0096 (14)	−0.0005 (13)
C12	0.0431 (16)	0.0383 (15)	0.0358 (16)	0.0025 (14)	−0.0143 (13)	0.0004 (13)
C13	0.0452 (17)	0.0378 (16)	0.062 (2)	−0.0073 (15)	−0.0266 (15)	0.0034 (14)
C14	0.0424 (18)	0.065 (2)	0.078 (2)	−0.0156 (18)	−0.0232 (16)	0.0116 (19)
C15	0.0419 (17)	0.067 (2)	0.064 (2)	0.0061 (17)	−0.0172 (15)	0.0063 (17)
N4	0.0287 (11)	0.0289 (11)	0.0388 (13)	−0.0024 (10)	−0.0077 (10)	0.0019 (10)
O1	0.0490 (11)	0.0291 (10)	0.0678 (14)	−0.0106 (9)	−0.0153 (10)	−0.0003 (9)
O2	0.0545 (12)	0.0307 (10)	0.0798 (16)	−0.0019 (10)	−0.0175 (11)	0.0179 (10)
S	0.0603 (5)	0.0458 (4)	0.0625 (5)	0.0053 (4)	−0.0086 (4)	0.0148 (4)
Cl1	0.0628 (5)	0.0437 (4)	0.0607 (5)	0.0027 (4)	−0.0044 (4)	0.0242 (4)
Cl2	0.0489 (4)	0.0382 (4)	0.0653 (5)	−0.0164 (3)	−0.0050 (4)	−0.0117 (3)
Cl3	0.0537 (4)	0.0620 (5)	0.0427 (4)	−0.0081 (4)	0.0166 (4)	0.0107 (4)
Cl4	0.0606 (5)	0.0437 (4)	0.0617 (5)	0.0095 (4)	0.0148 (4)	−0.0194 (4)
Cl5	0.0325 (3)	0.0753 (5)	0.0494 (4)	−0.0002 (4)	−0.0142 (3)	0.0020 (4)
Cl6	0.0611 (5)	0.0861 (6)	0.0308 (4)	−0.0023 (5)	0.0084 (4)	−0.0126 (4)

### Geometric parameters (Å, °)

**Table:**

C1—C9	1.519 (3)	C8—C9	1.320 (3)
C1—C10	1.551 (3)	C8—Cl3	1.695 (2)
C1—C2	1.554 (3)	C9—Cl4	1.689 (2)
C1—Cl1	1.741 (2)	C10—Cl6	1.761 (2)
C2—C3	1.508 (3)	C10—Cl5	1.763 (2)
C2—C6	1.542 (3)	C11—N4	1.464 (3)
C2—H2	0.9800	C11—C12	1.496 (3)
C3—O1	1.199 (3)	C11—H11A	0.9700
C3—N4	1.388 (3)	C11—H11B	0.9700
C5—O2	1.206 (3)	C12—C13	1.369 (3)
C5—N4	1.374 (3)	C12—S	1.714 (3)
C5—C6	1.504 (3)	C13—C14	1.409 (4)
C6—C7	1.558 (3)	C13—H13	0.9300
C6—H6	0.9800	C14—C15	1.344 (4)
C7—C8	1.516 (3)	C14—H14	0.9300
C7—C10	1.554 (3)	C15—S	1.701 (3)
C7—Cl2	1.749 (2)	C15—H15	0.9300
			
C9—C1—C10	99.40 (18)	C7—C8—Cl3	123.56 (17)
C9—C1—C2	107.31 (18)	C8—C9—C1	107.72 (19)
C10—C1—C2	100.37 (18)	C8—C9—Cl4	128.13 (19)
C9—C1—Cl1	116.18 (16)	C1—C9—Cl4	123.74 (18)
C10—C1—Cl1	116.20 (16)	C1—C10—C7	92.72 (17)
C2—C1—Cl1	115.16 (16)	C1—C10—Cl6	114.06 (17)
C3—C2—C6	104.81 (19)	C7—C10—Cl6	114.17 (17)
C3—C2—C1	112.73 (19)	C1—C10—Cl5	113.56 (17)
C6—C2—C1	103.33 (17)	C7—C10—Cl5	113.82 (16)
C3—C2—H2	111.8	Cl6—C10—Cl5	108.10 (12)
C6—C2—H2	111.8	N4—C11—C12	112.3 (2)
C1—C2—H2	111.8	N4—C11—H11A	109.1
O1—C3—N4	124.3 (2)	C12—C11—H11A	109.1
O1—C3—C2	127.9 (2)	N4—C11—H11B	109.1
N4—C3—C2	107.8 (2)	C12—C11—H11B	109.1
O2—C5—N4	124.2 (2)	H11A—C11—H11B	107.9
O2—C5—C6	127.5 (2)	C13—C12—C11	127.5 (2)
N4—C5—C6	108.2 (2)	C13—C12—S	111.32 (19)
C5—C6—C2	105.01 (19)	C11—C12—S	121.1 (2)
C5—C6—C7	113.76 (19)	C12—C13—C14	111.4 (2)
C2—C6—C7	102.84 (18)	C12—C13—H13	124.3
C5—C6—H6	111.6	C14—C13—H13	124.3
C2—C6—H6	111.6	C15—C14—C13	114.0 (3)
C7—C6—H6	111.6	C15—C14—H14	123.0
C8—C7—C10	99.38 (18)	C13—C14—H14	123.0
C8—C7—C6	107.48 (18)	C14—C15—S	111.3 (2)
C10—C7—C6	100.44 (17)	C14—C15—H15	124.4
C8—C7—Cl2	115.70 (16)	S—C15—H15	124.4
C10—C7—Cl2	116.52 (16)	C5—N4—C3	114.1 (2)
C6—C7—Cl2	115.18 (16)	C5—N4—C11	123.7 (2)
C9—C8—C7	107.86 (19)	C3—N4—C11	122.2 (2)
C9—C8—Cl3	128.35 (19)	C15—S—C12	91.96 (14)

### Hydrogen-bond geometry (Å, °)

**Table:**

*D*—H···*A*	*D*—H	H···*A*	*D*···*A*	*D*—H···*A*
C2—H2···O2^i^	0.98	2.54	3.064 (3)	113.
C6—H6···O2^i^	0.98	2.51	3.042 (3)	114.

Symmetry codes: (i) *y*−1/4, −*x*+7/4, *z*−1/4.
